# High-density Grid Mapping of Micro- and Macro-reentrant Left Atrial Arrhythmias

**DOI:** 10.19102/icrm.2021.120105S

**Published:** 2021-01-15

**Authors:** Jeremy P. Berman, Elaine Y. Wan, Deepak Saluja, Hasan Garan, Angelo Biviano

**Affiliations:** ^1^Electrophysiology Section, Division of Cardiology, Department of Medicine, Columbia University Vagelos College of Physicians and Surgeons, New York, NY, USA

**Keywords:** Atypical atrial flutter, focal atrial tachycardia, high-density grid, micro-reentrant atrial tachycardia

A 69-year-old woman with a history of heart failure with preserved ejection fraction and atrial fibrillation and flutter presented with recurrent palpitations. Prior radiofrequency ablation included left atrial pulmonary vein (PV) isolation, roof line, posterior mitral line, and right atrial cavotriscupid isthmus line. She was found to be in atypical atrial flutter consistent with a left atrial origin based on surface P-wave morphology [(−) I, aVL; (+) II, III, aVF; (+) V1–6] **(Figure 1)**.

Repeat radiofrequency ablation was performed, including three-dimensional electroanatomical and activation-sequence mapping using the Advisor™ HD Grid Mapping Catheter, Sensor Enabled™ to identify both micro-reentrant and macro-reentrant left atrial arrhythmias. First, the presenting rhythm was atypical atrial flutter with a cycle length (CL) of 275 ms, proximal-to-distal coronary sinus activation, and centrifugal spread consistent with a focal origin of the tachycardia from the left atrial roof adjacent to the right superior PV. Atrial electrograms at the proposed site of origin were low-amplitude, fractionated, and spanning nearly 100% of the tachycardia CL within the 1.3 × 1.3-cm high-density grid footprint **(Figures 2 and 3)**. Entrainment from this site yielded a postpacing interval minus the tachycardia CL of 5 ms, consistent with a micro-reentrant mechanism.

Next, radiofrequency ablation was delivered at the target site, resulting in transformation to a second atypical atrial flutter with a CL of 355 ms, which was determined to be a perimitral macro-reentrant flutter using high-density grid activation mapping **(Figure 4)** and entrainment. Formation of an anterior mitral line from the anterior mitral annulus to the anterior right superior PV terminated the arrhythmia **(Figure 5)**. Differential pacing maneuvers confirmed block across the anterior mitral isthmus line, roof line, and cavotricuspid isthmus and PV isolation. There were no immediate complications and the patient was discharged home the next day in sinus rhythm.

## Figures and Tables

**Figure 1:**
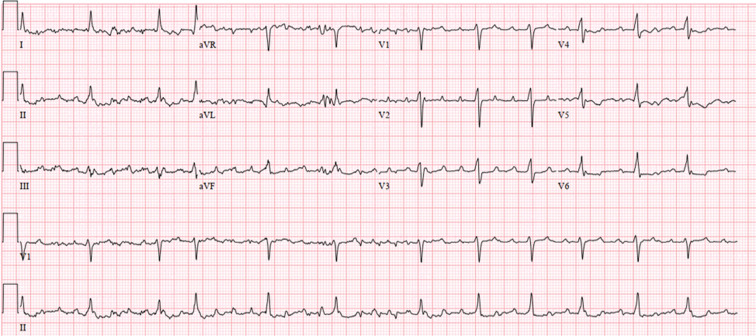
Twelve-lead electrocardiogram of the clinical tachycardia.

**Figure 2:**
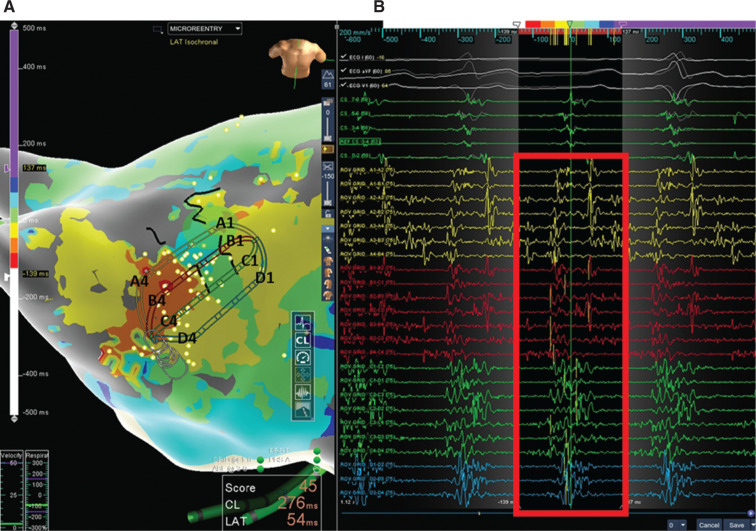
**A:** Left atrial isochronal activation map (modified anteroposterior view tilted inferiorly) of atypical atrial flutter no. 1 (CL: 275 ms) acquired with the Advisor™ HD Grid mapping catheter. Note the presence of six of eight isochrones (> 75% CL) within the 1.3 × 1.3-cm footprint of the Advisor™ HD Grid catheter located on the left atrial roof adjacent to the right superior PVs. The area between the black lines of block is a proposed isthmus for the micro-reentrant circuit with centrifugal spread. **B:** Corresponding electrograms from the Advisor™ HD Grid mapping catheter in this location showing low-amplitude, fractionated signals encompassing 75% to 100% of the CL.

**Figure 3:**
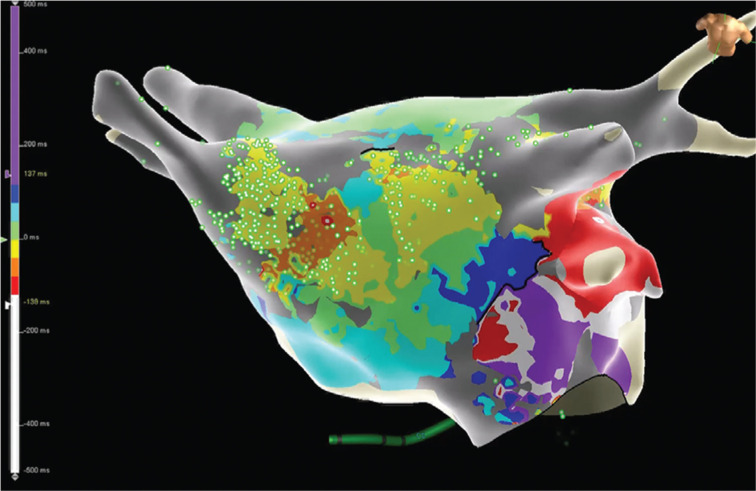
Still frame from the left atrial (modified anteroposterior view tilted inferiorly) sparkle map **(Video 1)** superimposed on an isochronal activation map of atrial tachycardia no. 1 acquired with the Advisor™ HD Grid mapping catheter showing the likely path of the micro-reentrant circuit on the left atrial roof adjacent to the right superior PV.

**Figure 4:**
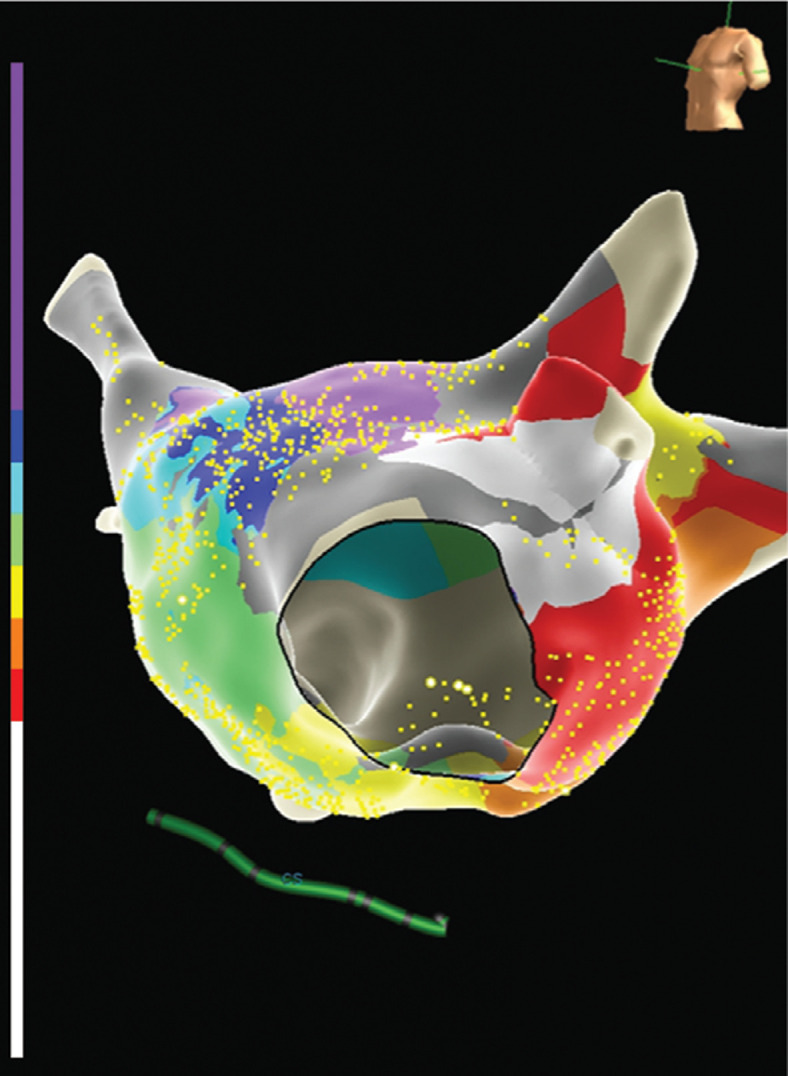
Left atrial (left anterior oblique view) activation map of atrial flutter no. 2 acquired with the Advisor™ HD Grid mapping catheter showing perimitral macro-reentrant flutter.

**Figure 5:**
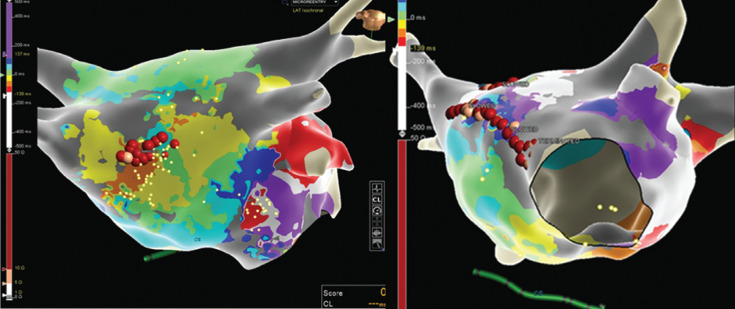
**A:** Left atrial (modified anteroposterior view tilted inferiorly) activation map of atrial flutter no. 1. The pink and red dots are the ablation lesions. **B:** Left atrial (left anterior oblique view) activation map of atrial flutter no. 2. The pink and red dots are the ablation lesions.

**Video 1.** Left atrial (modified anteroposterior view tilted inferiorly) sparkle map superimposed on an isochronal activation map of atrial tachycardia no. 1 acquired with the Advisor™ HD Grid mapping catheter showing the likely path of the micro-reentrant circuit on the left atrial roof adjacent to the right superior PV.

